# Pathogenesis of cerebral malaria—inflammation and cytoadherence

**DOI:** 10.3389/fcimb.2014.00100

**Published:** 2014-07-29

**Authors:** Janet Storm, Alister G. Craig

**Affiliations:** ^1^Department of Parasitology, Liverpool School of Tropical MedicineLiverpool, UK; ^2^Malawi Liverpool Wellcome Trust Clinical Research Programme (MLW), University of Malawi College of MedicineBlantyre, Malawi

**Keywords:** cerebral malaria, endothelium dysfunction, inflammation, histopathology, PfEMP1

## Abstract

Despite decades of research on cerebral malaria (CM) there is still a paucity of knowledge about what actual causes CM and why certain people develop it. Although sequestration of *P. falciparum* infected red blood cells has been linked to pathology, it is still not clear if this is directly or solely responsible for this clinical syndrome. Recent data have suggested that a combination of parasite variant types, mainly defined by the variant surface antigen, *P. falciparum* erythrocyte membrane protein 1 (PfEMP1), its receptors, coagulation and host endothelial cell activation (or inflammation) are equally important. This makes CM a multi-factorial disease and a challenge to unravel its causes to decrease its detrimental impact.

## Introduction

### Malaria

Malaria is a devastating infectious disease with an estimated 207 million cases and 627,000 deaths, mainly in children under 5 years of age in sub-Saharan Africa, in 2012 (World Health Organization, 2013). It is caused by the Apicomplexan parasite *Plasmodium*, of which *P. falciparum* is the most deadly of the species. Its asexual replication in the red blood cells (RBC) causes the pathology of the disease and during development it also modifies the host cell (White et al., 2014). In the trophozoite-stage, parasite proteins are exported to the surface of the RBC where they play a role in immune recognition and adherence to host endothelium (Kraemer and Smith, 2006; Chan et al., 2012; Craig et al., 2012a; Beeson et al., 2013), as well as other interactions with host cells (Pain et al., 2001; Fernandez and Wahlgren, 2002; Rowe et al., 2009). These parasite-host interactions are associated with the pathology of malaria and contribute to the development of severe malaria (SM). SM is defined by WHO as severe anemia, respiratory distress (or lactidosis) or coma, or a combination of these (Idro et al., 2005; Perkins et al., 2011; Shikani et al., 2012; Taylor et al., 2012). Most people suffer from uncomplicated malaria with only fever as a symptom, besides the presence of falciparum parasites in their blood. Many adults in endemic countries will encounter asymptomatic malaria, in which the infection is rapidly cleared by the host's immunological responses against the *P. falciparum* infected RBC (iRBC). However, only repeated *P. falciparum* infections give protection from disease, with sterile, anti-parasite immunity rarely achieved (Liehl and Mota, 2012; Spence and Langhorne, 2012; Portugal et al., 2013; Riley and Stewart, 2013).

### Cerebral malaria

Approximately 1% of *P. falciparum* infections results in CM with 90% of these cases in children in sub-Saharan Africa (W. H. World Health Organization, 2013). In adults in South-East Asia, CM accounts for 50% of the malaria deaths, as they not only suffer from encephalitis but also have multiple organ failure, which is absent in pediatric CM (Idro et al., 2005). More recently there has been an awareness of the burden of varying neurological deficit in survivors (Birbeck et al., 2010), increasing the impact on fragile economies. CM is clinically defined as having *P. falciparum* parasitaemia and unrousable coma, ruling out any other cause of coma, such as meningitis. Coma is assessed by verbal and motor responses and rated on either the pediatric Blantyre Coma Score (1 or 2 on a scale of 5) (Molyneux et al., 1989) or Glasgow Coma Score (<11 on a scale of 15) for adults (Teasdale and Jennett, 1974). Mortality is 15–20%, but 10–20% of survivors suffer from long-term neurological sequelae (Birbeck et al., 2010). The ultimate diagnosis is post-mortem through detecting brain hemorrhages and lesions in the brain with sequestered iRBC in the microvasulature. In a study of Malawian children clinically diagnosed with CM, 23% died of other causes than malaria (Taylor et al., 2004), demonstrating the difficulties faced by clinicians operating in an environment with a broad spectrum of severe disease.

The etiology of CM is not known. Although clearly linked to higher parasitaemias, these are not a prerequisite of CM (Gravenor et al., 1998) and it has been difficult to attribute specific parasite traits to the disease. Indeed, there has been considerable discussion about the relative roles of inflammation and cytoadherence (Berendt et al., 1994a; Clark and Rockett, 1994a,b; Ponsford et al., 2012; Cunnington et al., 2013a,b; Hanson et al., 2013; White et al., 2013), with both factors probably playing a role with varying influences in what is a variable clinical presentation (Grau and Craig, 2012). What does appear to be common is the presence of sequestered iRBC in the brains of people dying from this disease, although this does rely on post-mortem examination and therefore excludes observations on the majority of cases. In part this has been addressed by retinal examination (White et al., 2001; Maude et al., 2009), which provides a sensitive and specific indication of CM based on abnormal retinal characteristics that reflect some of the cerebral pathology and can therefore discriminate between coma caused by CM or other causes (MacCormick et al., 2014). Experimental CM, in which a *P. berghei* infection in mice causes CM, is frequently used to dissect the pathogenesis of CM. However, prominent sequestration of iRBC in the brain vasculature is not present and inflammatory processes are the main manifestation (de Souza et al., 2010; Craig et al., 2012b; Hansen, 2012). These studies are not included in this review, but they are discussed in two recent reviews on CM (Grau and Craig, 2012; Shikani et al., 2012).

## Pathogenesis of CM

### Histopathology studies

Sequestration of iRBC in the brain microvasculature is a hallmark of CM, with all autopsies showing the presence of iRBC causing congestion in the venules and capillaries, but sequestration is also detected in the brains of malaria patients who died of non-CM causes (NCM) (MacPherson et al., 1985; Maneerat et al., 1999; Taylor et al., 2004; Dorovini-Zis et al., 2011). However, the levels of sequestration and the percentage of sequestered vessels are significantly higher in the brains of CM sufferers and correlate to disease severity for both adults and children (MacPherson et al., 1985; Riganti et al., 1990; Maneerat et al., 1999; Dorovini-Zis et al., 2011; Ponsford et al., 2012). Sequestration is also detected in the lung, heart, kidney, skin and intestine and again this is higher in CM compared to NCM, but it is not as prominent as in the brain (MacPherson et al., 1985; Pongponratn et al., 1991; Seydel et al., 2006; Milner et al., 2013). The iRBC adhere to endothelium and the majority of *P. falciparum* are in the trophozoite or schizont stage, when they express parasite proteins on the surface of the RBC, such as the variant surface antigen PfEMP1.

In adults, cerebral sequestration is correlated with coma and earlier death, and it has been suggested that microvascular obstruction is key to the pathogenesis of coma (Ponsford et al., 2012). The main cause of coma is not known, and besides obstruction, a range of other mechanisms have been postulated, as reviewed by Idro et al. and Postels et al. (Idro et al., 2005; Postels and Birbeck, 2013). In pediatric CM, sequestration of iRBC is mostly (but not always) associated with microvascular pathology as demonstrated by endothelial damage and perivascular ring hemorrhages (Dorovini-Zis et al., 2011). Monocytes with malaria pigment and fibrin–platelet thrombi were also associated with iRBC sequestration and contribute to congestion (Dorovini-Zis et al., 2011). Whether these immune cells also contribute to microvascular congestion in adult CM is questionable (see section below), but obstruction by uninfected RBC has been detected in adult brain (Ponsford et al., 2012). Overall, microvascular congestion seems to leads to severe endothelial damage, causing disruption of the vessel wall, resulting in myelin and axonal damage and breakdown of the blood-brain barrier (BBB) (Brown et al., 1999; Dorovini-Zis et al., 2011). However, impairment of the BBB does not seem to occur to the same extent in adults (Medana and Turner, 2006; Renia et al., 2012; Polimeni and Prato, 2014), which could be due to the ongoing brain development in young children, which would make the BBB more susceptible (Hawkes et al., 2013).

### What is the primary cause of CM?

Whether sequestration, and therefore congestion, is the main cause of coma and CM has been debated for decades (Berendt et al., 1994b; Ponsford et al., 2012; Cunnington et al., 2013b; Hanson et al., 2013; White et al., 2013). The “school of sequestration” argue that:

1. The amount of sequestration and congestion correlates with disease severity.
2. Lactate production by anaerobic glycolysis, because of obstruction, is also correlated to the severity of the disease.
3. Impaired cytoadherence because of abnormal PfEMP1 distribution and reduced PfEMP1 expression on iRBC in haemopathies causes protection against severe disease (Fairhurst et al., 2012; Taylor et al., 2013).
4. Adjunctive therapies based on alternative causes of CM, like cytokine activation or hypovolemia, have not been proven effective (see table in White et al., 2013).

In contrast, the “school of cytokines” (Clark and Rockett, 1994a,b; Clark and Alleva, 2009) argue that:

1. Although P. vivax does not sequester in the microvasculature, it causes endothelial activation (Yeo et al., 2010) and encephalopathy and coma in children (Manning et al., 2012).
2. Healthy, malaria-tolerant children with high parasitaemia cannot reasonably be said to have parasites clogging their cerebral blood vessels (Clark and Alleva, 2009).
3. Pro-inflammatory TNF is associated with disease severity and is the main cytokine involved in CM.

Point (3) for the cytokine hypothesis is questionable with some studies showing a clear increase in TNF in CM, which is even higher in fatal cases in African children (Grau et al., 1989; Kwiatkowski et al., 1990; Sahu et al., 2013), but more recent studies have shown no correlation between TNF concentration and CM (Esamai et al., 2003; Armah et al., 2007; Thuma et al., 2011). Other cytokines have been implicated too (Hunt and Grau, 2003; Boeuf et al., 2012; Ioannidis et al., 2014), but the results of these studies are hard to interpret as it depends on which control group it is compared to; NCM, severe malarial anemia, other encephalitis, other febrile illnesses or even healthy individuals. In addition, patients often have other infections, especially African children, so the cytokine profile may not be a full reflection of the *Plasmodium* component of infection. The same is the case for the presence and role of immune cells in the cerebral vasculature. In post-mortem brain sections, sometimes neutrophils are seen (MacPherson et al., 1985), lymphocytes (Patnaik et al., 1994), monocytes/macrophages (Patnaik et al., 1994; Maneerat et al., 2000; Dorovini-Zis et al., 2011), or platelets (Pongponratn et al., 1985; Grau et al., 2003; Dorovini-Zis et al., 2011). These discrepancies in clinical observations may be the result of which part of the brain is used; cortex, cerebellum, brain stem, white or gray matter, how many hours after death the brain was sampled or to which control group the brain sections are compared.

Although the primary event in the development of CM still unknown, it is evident from recent work that endothelial activation plays a major role in the pathogenesis; whether this is caused by iRBC, parasite proteins (released after rupture of the RBC) (Gillrie et al., 2012), inflammatory cytokines or a combination of all of them is less clear.

### Endothelial dysfunction

Endothelial activation can be characterized by markers such as von Willebrand factor (Hollestelle et al., 2006), angiopoietein-1 and -2 (Lovegrove et al., 2009), and soluble endothelial receptors (Turner et al., 1998). Angiopoietein-1 stabilizes endothelium and angiopoietein-2 is its antagonist and is released by endothelial cells upon inflammatory stimuli and induces vascular permeability and up-regulation of endothelial receptors. Decreased Ang-1 or increased Ang-2 levels in serum, as measured by an increasing Ang-2/Ang-1 ratio, is associated with CM and can predict disease severity in adults and children (Conroy et al., 2009, 2010; Lovegrove et al., 2009; Jain et al., 2011; Prapansilp et al., 2013). Ang-1 and Ang-2 are regulated by nitric oxide, a signaling molecule in many processes, which is produced in the endothelium from L-arginine and causes vasorelaxation, vascular quiescence, down-regulation of endothelial adhesion molecules and reduces thrombosis (Bergmark et al., 2012). Nitric oxide bio-availability is reduced in SM and relates to fatal outcome and several groups have suggested that this could be linked to low L-arginine levels during malaria infection (Anstey et al., 1996; Lopansri et al., 2003; Yeo et al., 2007, 2008). Other markers of endothelial activation have been investigated, such as vascular endothelial growth factor, but the outcome is not always consistent between adults and children (Kim et al., 2011; Canavese and Spaccapelo, 2014).

Activated endothelium also over-expresses some of its receptors, which are subsequently shed into the plasma. *P. falciparum* infection up-regulates ICAM-1, V-CAM and E-selectin on cerebral microcvascular endothelium and some of these (e.g., ICAM-1) are co-localized with iRBC sequestration (Turner et al., 1994; Armah et al., 2005). However, there does not seem to be a specific correlation with CM as patients with uncomplicated malaria also have activated endothelium, although to a lesser extent (Turner et al., 1998; Rogerson et al., 1999). Unexpectedly, activation and systemic inflammation lasted for 4 weeks after diagnosis for all malaria cases, as measured by soluble ICAM-1, angiopoietein-2 and C-reactive protein (Moxon et al., 2014). Soluble ICAM-1 does seem to correlate with CM (Adukpo et al., 2013), but depending on the comparator control group (UM or other SM) it seems more strongly associated with SM (Cserti-Gazdewich et al., 2010; Erdman et al., 2011). Circulating endothelial microparticles are also associated with SM only in adults (Sahu et al., 2013) but do correlate with CM in children (Combes et al., 2004).

Endothelial dysregulation plays a role in the pathogenesis of SM, but this seems to be a general effect and is not confined to cerebral tissue alone. One of the problems in investigating vascular inflammation has been the difficulty in accessing cerebral tissue. Surrogate tissue, such as subcutaneous fat, has been used successfully to investigate microvascular endothelium (Wassmer et al., 2011; Moxon et al., 2013). This is easily obtained by biopsy and has some, but certainly not all, features of brain endothelium. These studies have shown that endothelial cells from children with CM show increased responsiveness to TNF and that the endothelium is changed by the presence of adherent iRBC, removing important control proteins involved in the coagulation/inflammation pathways.

A useful non-invasive tool in the clinical diagnosis of CM is retinopathy, in which the eye is used as a window into the brain. Sequestration and vascular damage can be visualized in the retina and the degree of damage correlates with the severity of microvascular brain damage and is a predictor of death (White et al., 2001; Maude et al., 2009; Beare et al., 2011), as is recently reviewed by MacCormick et al. (2014) The severity of retinopathy is also predictor for the neurocognitive problems that pediatric CM survivors suffer after recovering (Boivin et al., 2011).

### The role of the parasite

Why does sequestration of iRBC lead to vascular dysfunction in the brain? Sequestration of iRBC is also observed in other organs, but there seems to be no associated pathology. In CM, sequestration is the highest in the brain, but gastrointestinal and pulmonary sequestration is also present and in some cases very high. Unfortunately, not much histopathology has been performed on these tissues, but in pediatric CM only parasite sequestration and no other phenomena in the lungs was associated with CM (Milner et al., 2013). Furthermore, no hemorrhages, necrosis, abnormal architecture or inflammatory cells were detected in the gut (D. Milner, personal communication). Thus, do localized inflammatory responses to a *P. falciparum* infection have much greater effect on the cerebral vasculature? Or does the parasite itself play a significant role in combination with the host response?

Multiple *P. falciparum* genotypes are present in the human host, but only a subset of these sequester in the brain (Montgomery et al., 2006). Cytoadherence of iRBC to endothelial cell receptors is mediated by PfEMP1, one of the variant surface antigens of *Plasmodium falciparum* (Scherf et al., 2008; Pasternak and Dzikowski, 2009). Classification of the protein structure has recently been based on “domain cassettes” (DC), made up from combinations of the PfEMP1 Duffy Binding like (DBL) and Cysteine rich interdomain region (CIDR) subdomains (Rask et al., 2010). These DCs, along with other sequence signatures [e.g., PoLV (Bull et al., 2008)], have been associated with specific clinical outcomes, suggesting that particular domains of PfEMP1 play a role in pathology (Warimwe et al., 2012). Variants containing the conserved DC8 and DC13 are associated with SM in children (Claessens et al., 2012; Lavstsen et al., 2012) and are the main variant in parasites of children with CM in Benin (Bertin et al., 2013). The variants were identified by *in vitro* cytoadherence assays to human brain microvascular endothelial cells (Avril et al., 2012; Claessens et al., 2012), but they also bind avidly to endothelial cells of lung, heart and bone marrow, suggesting a common receptor (Avril et al., 2013). The PfEMP1-DC8 receptor was identified as Endothelial Protein C Receptor (EPCR) on brain endothelium and binding to EPCR, as well as ICAM-1, was shown to be associated with SM, including CM (Turner et al., 2013). ICAM-1 has long been implicated as an endothelial receptor in the brain and is associated with CM in some studies (Turner et al., 1994; Ochola et al., 2011). Recently, seven new receptors for PfEMP1 were identified that seem to be expressed on brain endothelium, but no correlation with disease severity has been shown yet (Esser et al., 2014).

In binding to EPCR the iRBC blocks the conversion of inactive protein C to activated protein C, potentially contributing to localized endothelial activation. In a separate study it was shown that adhesion of iRBC to endothelium reduced the levels of EPCR and its coagulation partner thrombomodulin on endothelium and suggested that the relatively low expression of these receptors on brain endothelium compared to other tissues would make the brain particularly susceptible to inflammation caused by reduced control of thrombin production. Taken together both of these studies suggest an important role for localized coagulation/inflammation related pathology in CM. (Moxon et al., 2013; Aird et al., 2014; Angchaisuksiri, 2014).

**Figure 1:**
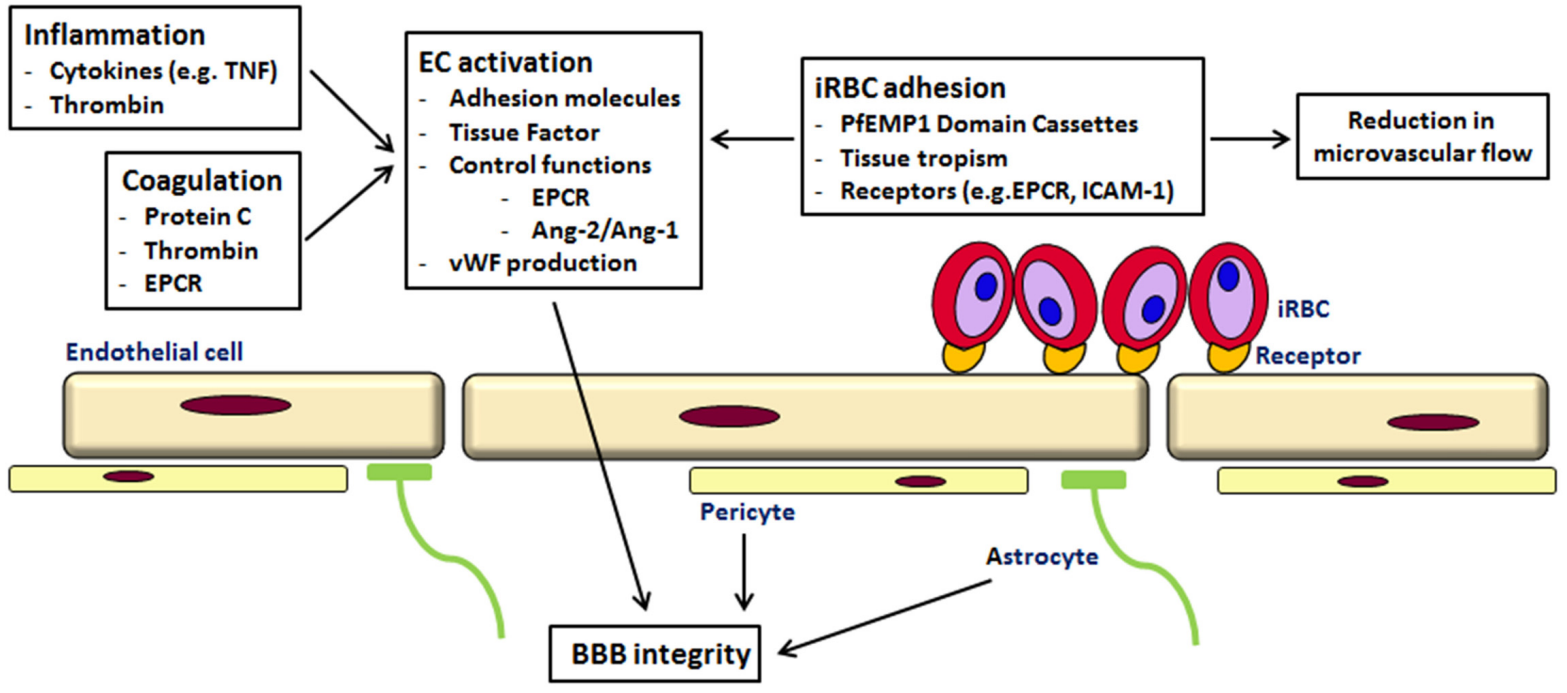
**Cerebral malaria—a multi-component disease**. Cytoadherence of iRBC of the PfEMP1-DC8 and DC13 variants to endothelial receptors (like EPCR and ICAM-1) leads to sequestration and a reduction in microvascular flow. Binding to EPCR prevents activation of protein C, normally an inhibitor of thrombin generation. Increased thrombin affects various signaling pathways leading to loss of endothelial barrier function, increased TNF, Ang2/Ang1 ratio and von Willebrand factor (for a detailed description see Moxon et al., 2013). iRBC in the circulation and the rupture of iRBC elicits an immune response and the production of cytokines, Via various signaling pathways this leads to inflammation, an increased expression of endothelial receptors and therefore shedding of soluble endothelial receptors and ultimately to endothelial damage (see Miller et al., 2013). The leakage into the perivascular space affects astrocytes and pericytes leading to BBB impairment.

As suggested by others, the pathogenesis of CM is likely to be a multi-factorial process, with sequestration, inflammation and endothelial dysfunction in the microvasculature of the brain leading to coma (see Figure 1) (van der Heyde et al., 2006; Milner, 2010; Grau and Craig, 2012; Shikani et al., 2012; Cunnington et al., 2013b). However, the recent findings of the involvement of coagulation and cytoadherence of particular parasite variants to specific receptors have to be taken into account. So perhaps the reason why brain microvascular endothelium is more susceptible to a *P. falciparum* infection than other tissues is the occurrence of the “correct” combination of parasite variant, endothelial receptors, local inflammation, coagulation and endothelial activation Thus, future work may need to focus not on single attributes associated with disease but more complicated interactions based on both host and pathogen characteristics. This has recently been shown in children with CM; the expression of a parasite PfEMP1 variant (group A) and endothelial activation, as measured by angiopoitein-2 concentration, were independently associated with coma (Abdi et al., 2014).

## Diagnosis and treatment of CM

### Biomarkers for CM

The emphasis on just one feature of CM development probably explains why identified biomarkers, to diagnose CM in an early stage or to predict death, have shown only moderate sensitivity and specificity. Up to 2011 there was no single reliable biomarker for CM, but potential markers, including the Ang2/Ang1 ratio, were identified, as reviewed by Lucchi (Lucchi et al., 2011). However, combining multiple host markers of inflammation and endothelial activation can predict mortality accurately in children with SM (Erdman et al., 2011). Histidine-rich protein 2 (HRP2) seems a promising candidate, measuring total parasite biomass, peripheral and sequestered. Its high concentration on admission is prognostic for the risk of death due to SM, including CM (Dondorp et al., 2005; Hendriksen et al., 2012; Fox et al., 2013) and furthermore the concentration is elevated in children with retinopathy compared to children without retinopathy, discriminating between coma caused by CM or other infections. (Seydel et al., 2012; Kariuki et al., 2014). Recently, proteomics has been utilized to screen for potential biomarkers and it may also identify pathways which relate to the pathogenesis of CM (Burte et al., 2012; Gitau et al., 2013; Bachmann et al., 2014).

### Therapeutic approaches

Treatment of CM with intravenous artesunate (the WHO recommendation) is not sufficient to prevent all deaths, although it has shown a significant reduction compared to quinine. To further reduce mortality and neurological sequelae in survivors, adjunctive therapies are required. As reviewed by Higgins et al not one immuno-modulatory intervention tested till 2011 had a clear benefit and often adverse events were reported (Higgins et al., 2011). Meanwhile, two other randomized controlled trials have been reported; vitamin A in Ugandan children (Mwanga-Amumpaire et al., 2012) and levamisole in adults with SM in Bangladesh (Maude et al., 2014), but no benefit was recorded in both studies. Clinical trials with erythropoietin and inhaled nitric oxide are apparently ongoing. For L-arginine, the precursor of nitric oxide, a randomized pilot has shown that it was safe, but it did not improve nitric oxide bioavailability (Yeo et al., 2013). Furthermore, therapies that prevent seizures during coma and neurological damage in survivors, such as fosphenytoin, also do not have an effect (Gwer et al., 2013). All these interventions were based on one of the attributes of the pathogenesis of CM and it will be more beneficial to target multiple mechanisms, as outlined above.

## Summary

The picture that is emerging for CM is complicated, both in the variability of its presentation between adults and children, and even within children, as well as the mechanisms that cause pathology. Our understanding of the events taking place at the microvascular interface is improving and this greater knowledge will hopefully allow us to design therapeutic interventions that can act to prevent severe symptoms as well as to reverse or reduce mortality and morbidity in established infections.

### Conflict of interest statement

The authors declare that the research was conducted in the absence of any commercial or financial relationships that could be construed as a potential conflict of interest.
